# The impact of social support on the quality of sports participation among individuals with disabilities: the chain mediating effects of psychological capital and self-esteem

**DOI:** 10.3389/fpsyg.2025.1662636

**Published:** 2025-11-05

**Authors:** Jiahui Peng, Li Cao, Feixuan Li, Deqiao Zhou

**Affiliations:** ^1^College of Physical Education and Sport Science, Qufu Normal University, Jining, China; ^2^School of Physical Education, Shandong Normal University, Jinan, China; ^3^School of Physical Education, Shandong University, Jinan, Jinan, China

**Keywords:** social support, individuals with disabilities, quality of sports participation, psychological capital, self-esteem, chain mediating effect

## Abstract

**Background:**

Individuals with disabilities possess the right to equally participate in cultural, recreational, and sporting activities. Enhancing the quality of sports participation among this population is therefore of significant importance for promoting social equity. Grounded in Conservation of Resources Theory and the Social Cognitive Framework, this study focuses on the sequential transmission pathway involving psychological capital and self-esteem. It aims to elucidate the chain mediation mechanism through which social support influences the quality of sports participation among individuals with disabilities, thereby providing a theoretical foundation and practical targets for optimizing sports policies for this group.

**Methods:**

Employing a stratified random sampling method, a cross-sectional questionnaire survey was conducted with 632 individuals with disabilities across 12 cities in 6 provinces spanning Eastern, Central, and Western China. Data collected were analyzed using SPSS for descriptive statistics, correlation analysis, and regression analysis. The bias-corrected bootstrap method (5,000 resamples) was utilized to test the chain mediating effects.

**Results:**

(1) Social support significantly and positively predicted the quality of sports participation among individuals with disabilities (Total effect = 0.786, 95% CI [0.680, 0.891]), with the direct effect accounting for 49.87%. (2) Both psychological capital (*β* = 0.478) and self-esteem (*β* = 0.154) independently and positively predicted the quality of sports participation. (3) The total mediating effect of psychological capital and self-esteem was significant (Effect = 0.394, 95% CI [0.313, 0.484]), accounting for 50.13% of the total effect. Among the specific paths, “Social Support → Psychological Capital → Quality of Sports Participation” contributed the most (Effect = 0.294, Proportion = 37.41%). The chain path “Social Support → Psychological Capital → Self-Esteem → Quality of Sports Participation” was also significant (Effect = 0.025, Proportion = 3.18%).

**Conclusion:**

Social support not only directly enhances the quality of sports participation among individuals with disabilities but also facilitates a transformation from external empowerment to internal drive through the chain psychological mechanism of “Psychological Capital → Self-Esteem.” Theoretically, this study verifies the hierarchical progressive effect of positive psychological resources within the “Environment-Cognition-Behavior” pathway, deepening the psychological dynamic model of sports participation for individuals with disabilities. Practically, it suggests that improving participation quality requires the simultaneous construction of a multi-level social support network and the development of targeted integrated intervention programs focusing on psychological capital and self-esteem, to achieve a synergistic leap from participation “quantity” to experiential “quality.”

## 1 Introduction

Sports participation among individuals with disabilities, as a vital pathway for promoting social inclusion and individual development, has emerged as a critical issue of international concern. The United Nations Convention on the Rights of Persons with Disabilities explicitly emphasizes the right of persons with disabilities to participate equally in cultural, recreational, and sporting activities, recognizing this as a key indicator of societal progress. China has also actively responded to this global trend by strengthening the provision of sports public services and prioritizing the quality of sports participation among individuals with disabilities through its “14th Five-Year Plan for the Protection and Development of Persons with Disabilities.” Enhancing the quality of sports participation for this population is not only essential for individual health rehabilitation but also represents a strategic pathway toward social integration and psychological empowerment (Kong, 2023).

At the physiological level, exercise interventions can activate the functional compensatory potential of impaired body regions (Ravesloot et al., 2022). Psychologically, achievement experiences in sports can rebuild self-efficacy and alleviate negative emotions (Niedbalski, 2018). Socially, inclusive sports activities help dismantle social isolation (Al Harthy et al., 2025).

As a socially vulnerable group, individuals with disabilities rely on social support not only for their overall well-being but also for the quality of their sports participation. However, existing research has yet to clarify the impact of social support on the quality of sports participation among individuals with disabilities or its underlying mechanisms (Ayyildiz et al., 2024). This study aims to construct a chain mediation model—“Social Support(SS) → Psychological Capital(PC) → Self-Esteem(SE) → Quality of Sports Participation(QSP)”—to explore the intrinsic psychological mechanisms through which external support translates into sports participation behavior.

Theoretically, this research integrates dual perspectives from positive psychology and sports psychology to validate the sequential roles of PC and SE, thereby deepening the understanding of the “Environment-Cognition-Behavior” pathway. Practically, it provides targeted intervention evidence for optimizing sports policies for individuals with disabilities. Should the chain mediation effect prove significant, it would highlight the need to simultaneously enhance SS networks and psychological empowerment programs, facilitating a shift from quantitative participation to qualitative experience.

## 2 Literature review and research hypotheses

### 2.1 Social support and quality of sports participation among individuals with disabilities

SS theory emphasizes that emotional, instrumental, informational, and appraisal support from family, friends, community, and professionals provides crucial resources for individuals to cope with challenges, buffers stress, and enhances their sense of well-being and efficacy (Blaney et al., 1991). For individuals with disabilities, participation in sports activities often involves unique physiological, psychological, and environmental barriers, such as functional limitations, inadequate accessibility in venues, social prejudice, or low self-efficacy (Li and Zhu, 2023). In this context, adequate and appropriate SS—such as encouragement and assistance from family, acceptance and companionship from peers, professional guidance from coaches, and accessible environments and participation opportunities provided by the community—can directly or indirectly lower the threshold for sports participation, enhance feelings of safety and comfort during participation, strengthen confidence and motivation to persist in exercise, and fulfill social and belonging needs (Hill et al., 2025). Therefore, the combined effect of various elements of SS is expected to directly promote the frequency, persistence, engagement level, and overall satisfaction of sports participation among individuals with disabilities, thereby significantly improving its quality. Existing research has confirmed the positive effect of SS on physical exercise behaviors in the general population and specific groups (Shang et al., 2025; Zi Zun L. T. C. et al., 2024). Although studies focusing specifically on the QSP among individuals with disabilities are relatively scarce, preliminary evidence also supports a positive association between SS and the level of sports activity participation in this population (Mira et al., 2023). Based on this, the following hypothesis is proposed:

*H1*: SS can positively predict the QSP among individuals with disabilities.

### 2.2 The mediating effect of psychological capital

PC refers to a set of positive psychological characteristics exhibited by individuals during their growth and development (Peng et al., 2024). As a positive psychological resource, PC exerts a broad influence on individuals’ attitudinal tendencies and behavioral patterns (Mao and Xie, 2013). Previous research has found a significant positive correlation between SS and PC, with the level of SS significantly predicting the level of PC (Cheng and Gao, 2019). Furthermore, findings indicate that a high level of PC directly empowers individuals to overcome physical, environmental, and psychological barriers encountered in sports participation, promoting more persistent, engaged, skillfully developed, and subjectively positive participation behaviors (Yu et al., 2025). Thus, SS may not only directly affect participation behavior and its quality but also indirectly and more profoundly enhance the depth, breadth, persistence, and sense of achievement in sports participation by cultivating and strengthening the intrinsic positive PC of individuals with disabilities. Based on this, the following hypotheses are proposed:

*H2*: SS can positively predict the PC of individuals with disabilities.

*H3*: PC can positively predict the QSP among individuals with disabilities.

*H4*: PC mediates the effect of SS on the QSP among individuals with disabilities.

### 2.3 The mediating effect of self-esteem

As an external resource system, SS influences the QSP by reinforcing internal psychological mechanisms. SE, as a core component of self-worth cognition, is expected to function as a conduit in this pathway. According to the SS buffering model and identity theory, perceived instrumental and emotional support among individuals with disabilities significantly enhances their self-efficacy and identity, thereby positively predicting increased levels of SE (Zi Zun L. B. et al., 2023). Based on the three basic psychological needs—autonomy, competence, and relatedness—proposed by self-determination theory, a high level of SE directly promotes the depth and persistence of sports participation among individuals with disabilities (Peng et al., 2025). SE, as a stable evaluation of one’s own worth, can enhance psychological resilience when facing athletic challenges, reduce the probability of activity withdrawal, strengthen goal orientation and flow experiences during exercise, and consequently improve multidimensional indicators of participation quality (Zi Zun D. Z. H. et al., 2023). Previous research indicates that the SE of individuals with disabilities is often constrained by the dual pressures of social stigma and physical dysfunction. Sports participation, as an empowering activity, relies heavily on the establishment of a positive self-concept for quality enhancement (Zi Zun and Wu, 2023), providing theoretical justification for the mediating role of SE.

Simultaneously, SS networks continuously modify an individual’s self-schema by providing affirming feedback and opportunities for achievement experiences, fostering a positive identity as a “capable participant” (Long et al., 2025). This reconstructed state of SE, in turn, stimulates stronger intrinsic motivation for exercise, buffers the negative impact of external barriers, and ultimately optimizes the structural dimensions of QSP. Based on this, the following hypotheses are proposed:

*H5*: SS can positively predict the SE of individuals with disabilities.

*H6*: SE can positively predict the QSP among individuals with disabilities.

*H7*: SE mediates the relationship between SS and the QSP among individuals with disabilities.

### 2.4 The chain mediating effect of psychological capital and self-esteem

According to Conservation of Resources theory, the accumulation and activation of PC directly shape an individual’s fundamental evaluation of their existential worth (Han et al., 2024). Specifically: Self-efficacy, as the cognitive-motivational dimension of PC, enables individuals to develop stable beliefs in their ability to successfully cope with specific situations. The sense of mastery derived from successful experiences is continuously internalized into an affirmative assessment of self-worth through the chain of “competence confirmation → achievement attainment → value reinforcement,” thereby providing a solid cognitive foundation for SE. Optimism, as the affective-motivational dimension, leads individuals to hold positive expectations about future outcomes and to attribute positive events to internal, stable, and global causes. This positive attributional style effectively buffers the negative impact of failures on self-image, maintaining the stability of SE. Resilience, as the dynamic adaptation dimension, enables individuals not only to recover quickly from adversity but also to achieve positive adaptation through proactive adjustments in cognition and behavioral strategies (Masten, 2001). Furthermore, the “recovery-growth” process itself constitutes repeated validation of an individual’s coping efficacy and intrinsic value, directly enhancing the depth of SE. Hope, as the goal-oriented dimension, propels individuals to set and pursue meaningful goals through the synergy of “pathways thinking” (identifying ways to achieve goals) and “agency thinking” (motivation to use those pathways) (Zi Zun, 2021). The process of goal pursuit and its attainment provides objective evidence of self-competence, offering continuous reinforcing feedback for SE.

**Figure 1:**
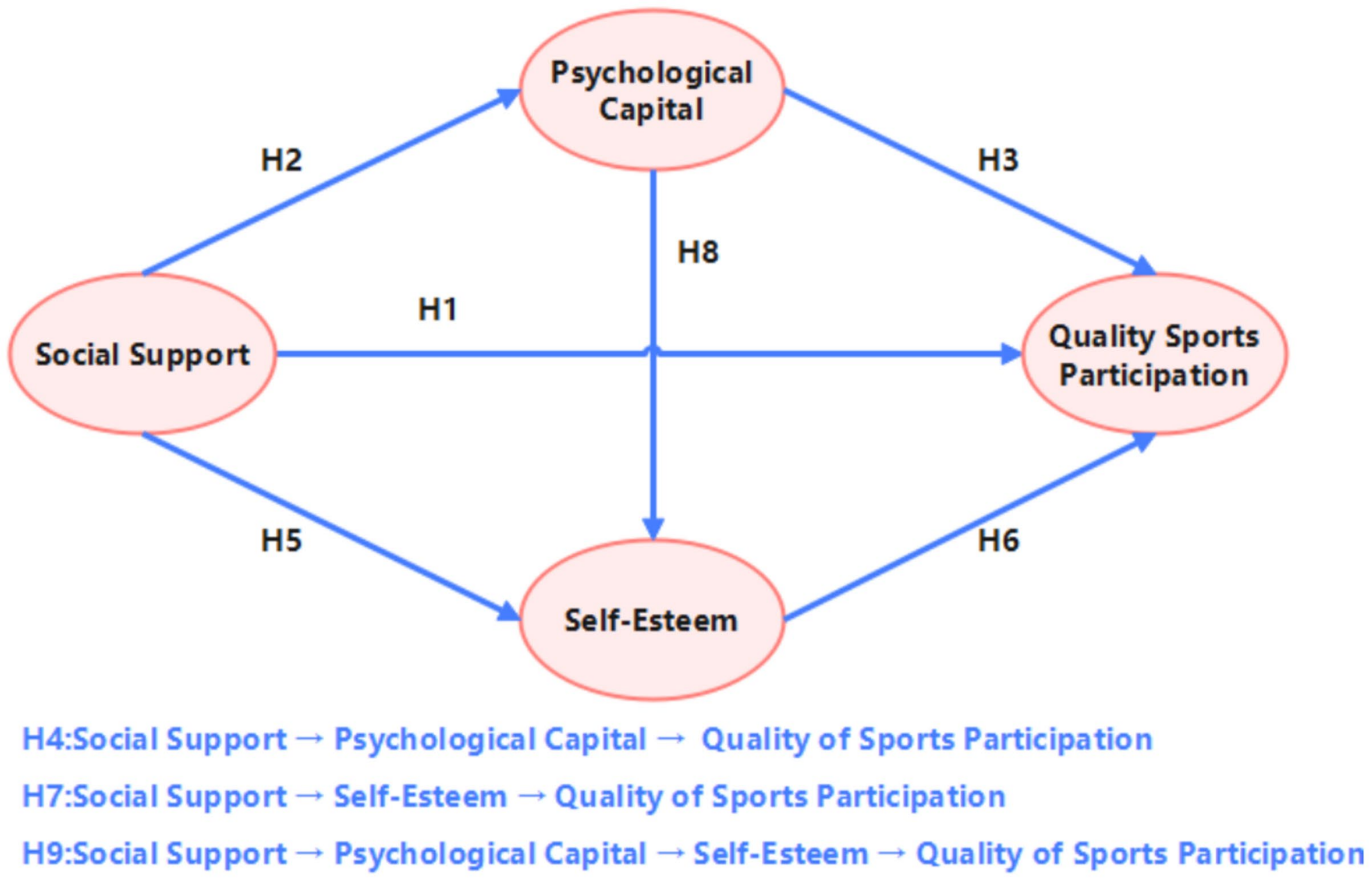
Hypothesized model.

Evidently, SE and PC are closely related; PC can positively predict an individual’s SE, meaning individuals with high PC tend to have higher levels of SE (Zi Zun and Kuang, 2017). Research also indicates a significant correlation between positive PC and SE, which can predict exercise motivation (Figure 1; Zi Zun D. Y. Q. et al., 2024). Based on this, the following hypotheses are proposed:

*H8*: PC can positively predict SE.

*H9*: PC and SE jointly mediate the relationship between SS and the QSP among individuals with disabilities.

## 3 Research methods

### 3.1 Participants

The focus of this study was to examine the impact of SS on the QSP among individuals with disabilities and the mediating roles of PC and SE. The required sample size was estimated using G^*^Power 3.1 software. With *α* set at 0.05, statistical power (1 − β) at 0.99, and a medium effect size (*f*^2^ = 0.15) specified for multiple regression analysis, the calculation indicated a minimum required sample size of 203.

A stratified random sampling method was employed. Six provinces were selected from the Eastern, Central, and Western regions of China. Within each province, two cities were randomly chosen, resulting in a total of 12 cities. Individuals with disabilities within these cities served as participants. Questionnaires were distributed randomly using both online and paper-based formats within each city. A total of 230 paper questionnaires and 420 electronic questionnaires were distributed. After excluding 28 invalid questionnaires (due to patterned responses, incomplete responses, or online completion times under 2 min), 632 valid questionnaires were retained, yielding a valid response rate of 97.2%.

During the process of administering the questionnaires, we implemented multi-dimensional and individualized support strategies to ensure that participants with sensory or motor impairments could participate in the study smoothly, autonomously, and effectively. At the questionnaire design stage, we thoroughly considered the practical needs of individuals with different types of disabilities. For those with visual impairments, we provided large-print versions of the materials and voice-assisted reading, with Braille versions available upon request. For participants with hearing impairments, instructions and items were clearly presented in written form, supplemented by sign language interpretation or visual aids when necessary to enhance comprehension. During data collection, we adapted the response methods flexibly according to participants’ physical functional abilities. For example, individuals with upper limb mobility impairments were permitted to complete the questionnaire through oral responses, eye-tracking devices, or with the assistance of a helper for transcription. Throughout this process, we emphasized the principle of neutrality for assistants, ensuring that they provided only operational support without influencing the content of responses. Furthermore, prior to beginning the questionnaire, we engaged in thorough communication with each participant to clarify their specific needs and preferences. During the completion process, we maintained a patient, respectful, and supportive attitude, fostering a safe and comfortable environment for expression. This approach not only helped safeguard data quality but also upheld participants’ autonomy and dignity. These comprehensive measures not only enhanced the validity of the collected responses but also reflected the study’s commitment to inclusivity and ethical considerations in its methodological design.

The final sample size exceeded the minimum requirement calculated by G^*^Power. The specific demographic characteristics of the sample are presented in Table 1.

**Table 1:** Distribution of demographic characteristics of the participants.

Variable	Category	Frequency	Effective percentage
Gender	Male	301	47.6
	Female	331	52.4
Region	East	214	33.9
	Central region	223	35.3
	West	195	30.9
Age	20 ~ 30	166	26.3
	31 ~ 40	161	25.5
	41 ~ 50	143	22.6
	51 ~ 60	162	25.6
Urban and rural areas	Rural areas	317	50.2
	City	315	49.8

### 3.2 Measurement instruments

#### 3.2.1 Social support scale

The SS Rating Scale, developed by Chinese scholar Xiao (1994), was used to measure SS. It comprises 10 items across three dimensions: Objective Support, Subjective Support, and Utilization of Support. The SSRS has undergone multiple validations and is suitable for assessing “SS” within the Chinese context. Responses were recorded on a 4-point Likert scale. The total score ranges from 12 to 66, with higher scores indicating a higher level of perceived SS. In this study, the scale demonstrated good structural validity: KMO measure of sampling adequacy was 0.897, and Bartlett’s test of sphericity was significant (*χ*^2^ = 1187.633, *p* < 0.001). The internal consistency reliability was good (Cronbach’s *α* = 0.802).

#### 3.2.2 Quality of sports participation scale

The “Physical Activity Rating Scale,” revised by Chinese scholar Liang (1994), was employed to assess the QSP. This scale evaluates exercise volume based on three dimensions: exercise intensity, exercise frequency, and exercise duration. The PARS-3 has been validated multiple times and is appropriate for measuring “QSP” in China. Responses were recorded on a 5-point Likert scale. The total exercise volume score was calculated using the formula: Exercise volume = Exercise frequency × (Exercise duration − 1) × Exercise intensity. Scores range from 0 to 100, serving as the indicator for QSP. In this study, the scale showed good structural validity (KMO = 0.771; Bartlett’s test: *χ*^2^ = 349.985, *p* < 0.001) and acceptable internal consistency reliability (Cronbach’s *α* = 0.740).

#### 3.2.3 Psychological capital scale

The Positive PC Questionnaire, adapted and revised into Chinese by scholars Zhang et al. (2010) based on the PC Questionnaire, was used to measure PC levels. It consists of 26 items, including 5 reverse-scored items. The Chinese Positive PC Questionnaire has been validated multiple times and is suitable for the Chinese context. Responses were recorded on a 7-point Likert scale. Total scores range from 26 to 182, with higher scores indicating higher levels of PC. The scale exhibited excellent structural validity (KMO = 0.978; Bartlett’s test: *χ*^2^ = 6958.312, *p* < 0.001) and excellent internal consistency (Cronbach’s α = 0.946) in this study.

#### 3.2.4 Self-esteem scale

The Rosenberg Self-Esteem Scale, originally developed by Rosenberg (1965) and later translated and revised for Chinese populations by scholars including Wang Mengcheng, was used. The Chinese version contains 10 items. Considering cultural differences between China and the West and referencing prior research (Huang and Chen, 2025), items 8 and 9 were omitted, resulting in an 8-item scale. The decision to exclude two items from the scale in this study was based on several methodological considerations. First, from the perspective of measurement validity, although the original scale demonstrates robust psychometric properties, certain items may exhibit limited cultural adaptability in cross-cultural research contexts, potentially leading to semantic interpretation biases and thereby compromising the precision of construct measurement. Second, given the specific characteristics of the population under investigation, the focal group in this study displays distinct tendencies in the manifestation of self-esteem. The retained items were deemed more effective in capturing the core features of the target construct, whereas the excluded items showed low conceptual correlation with others in preliminary tests, suggesting that their inclusion might introduce measurement noise. Furthermore, adhering to the principle of parsimony in methodology, removing the two items with relatively weak factor loadings helps preserve the fundamental structure of the scale while enhancing its internal consistency. This decision was supported by rigorous item analysis and validity assessments, aligning with psychometric standards for instrument refinement and ensuring that the data align well with the theoretical framework. Ultimately, this approach provides a more reliable measurement foundation for in-depth exploration of the research questions. This revised Chinese RSES has been validated and is appropriate for assessing “SE” in China. Responses were recorded on a 4-point Likert scale (1 = “Strongly Agree” to 4 = “Strongly Disagree”). All 8 items were positively scored, with higher total scores indicating higher levels of SE. In this study, the scale demonstrated good structural validity (KMO = 0.829; Bartlett’s test: *χ*^2^ = 702.603, *p* < 0.001) and acceptable internal consistency (Cronbach’s *α* = 0.706).

## 4 Research results

### 4.1 Common method bias test

Common method bias was assessed using statistical procedures. Harman’s single-factor test was conducted by performing exploratory factor analysis (EFA) on all measurement items. The results indicated that the first unrotated principal component explained 26.493% of the total variance, which is below the critical threshold of 40%, suggesting that severe common method bias was not present in the data. Although a single factor did not account for the majority of the variance, additional measures were implemented during the questionnaire design phase to control for potential bias: (1) Items for independent variables and dependent variables were separated in the sequence; (2) Scales from diverse sources were utilized, and item wording was kept clear and unambiguous; (3) Anonymous responses were collected. For further robustness, supplementary verification using the unmeasured latent method factor approach or marker variable technique could be conducted in subsequent analyses. In summary, the quality of the data met the fundamental requirements for statistical analysis.

### 4.2 Correlation analysis of research variables

Descriptive statistics and Pearson product–moment correlation coefficients for the core variables are presented in Table 2. As shown in the table, the mean scores for SS, PC, and SE were all at a moderately high level, indicating that the participants perceived reasonably adequate levels of these psychosocial resources. However, the mean score for the QSP was relatively low, suggesting insufficient QSP among the participant group.

**Table 2:** Correlation test.

Variable	*B*	SE	SS	QSP	PC	SE
SS	3.143	0.591	1			
QSP	1.203	0.927	0.502^***^	1		
PC	3.136	0.747	0.397^***^	0.637^***^	1	
SE	3.100	0.537	0.427^***^	0.449^***^	0.399^***^	1

^*^p < 0.05; ^**^p < 0.01; ^***^p < 0.001, the same below.

Correlation analysis revealed statistically significant positive correlations among all variables. Specifically: SS showed a moderately significant positive correlation with the QSP, and significant positive correlations with both PC and SE. The QSP exhibited a strong positive correlation with PC and a moderate positive correlation with SE. A significant positive correlation was also found between PC and SE. This overall pattern of the correlation matrix aligns with theoretical expectations. The moderate magnitudes of the correlations among the variables provide preliminary statistical support for the subsequent test of the chain mediating effects involving PC and SE.

### 4.3 Multiple linear regression analysis

Multiple linear regression analysis was employed to examine the joint predictive effects of SS, PC, and SE on the QSP among individuals with disabilities. As presented in Table 3, the overall regression model was statistically significant (*F* = 206.634, *p* < 0.001).

**Table 3:** Results of multiple linear regression analysis predicting quality of sports participation.

Variable	M	SD	*β*	*t*	Tolerance	VIF
Constant	−2.712	0.18		−15.051		
SS	0.385	0.051	0.246	7.567	0.757	1.322
PC	0.592	0.04	0.478	14.882	0.778	1.286
SE	0.265	0.056	0.154	4.716	0.755	1.324

The results indicated that, after controlling for other variables in the model: SS demonstrated a significant positive predictive effect on the QSP (*β* = 0.246, *t* = 7.567, *p* < 0.001). This signifies that for every one-unit increase in SS, the QSP is expected to increase significantly by 0.385 units. PC emerged as the strongest predictor in the model (*β* = 0.478, *t* = 14.882, *p* < 0.001). A one-unit increase in PC was associated with a significant expected increase of 0.592 units in the QSP. SE also showed a significant positive predictive effect (*β* = 0.154, *t* = 4.716, *p* < 0.001). The constant term (intercept) of the model was statistically significant (*B* = −2.712, SE = 0.180, *t* = −15.051, *p* < 0.001).

Diagnostic metrics indicated no severe multicollinearity issues: the Tolerance values for all independent variables were greater than 0.7, and the Variance Inflation Factors (VIF) were all well below the critical threshold of 5. This confirms the stability of the regression coefficient estimates. In summary, SS, PC, and SE were all confirmed as significant positive factors enhancing the QSP among individuals with disabilities. This finding provides essential prerequisite support for the subsequent investigation of the chain mediation pathways among these variables.

### 4.4 Mediation effect test

The chain mediating roles of PC and SE in the relationship between SS and the QSP among individuals with disabilities were tested using the bias-corrected bootstrap method (with 5,000 resamples). The specific path effect sizes and their significance are detailed in Table 4.

**Table 4:** Results of mediation effect path analysis.

Effect	Path	Effect size	Standard error	LLCL	ULCL	Percentage of total effect
Total effect	Direct path	0.786	0.054	0.680	0.891	100.00
Direct effect		0.392	0.051	0.292	0.492	49.87
Total indirect effect		0.394	0.043	0.3127	0.484	50.13
Indirect effect	Pathway 1	0.294	0.042	0.217	0.384	37.41
	Pathway 2	0.075	0.027	0.026	0.133	9.54
	Pathway 3	0.025	0.010	0.008	0.048	3.18

The results revealed that the total effect of SS on the QSP was 0.786 (SE = 0.054), with a 95% confidence interval (CI) of [0.680, 0.891], excluding zero. This indicates a significant positive overall impact of SS on QSP. The direct effect of SS on QSP was 0.392 (SE = 0.051), significant (95% CI [0.292, 0.492]), accounting for 49.87% of the total effect. Furthermore, the total indirect effect mediated through PC and SE was 0.394 (SE = 0.043), also significant (95% CI [0.313, 0.484]), accounting for 50.13% of the total effect. This confirms the existence of significant mediation effects with substantial explanatory power.

**Figure 2:**
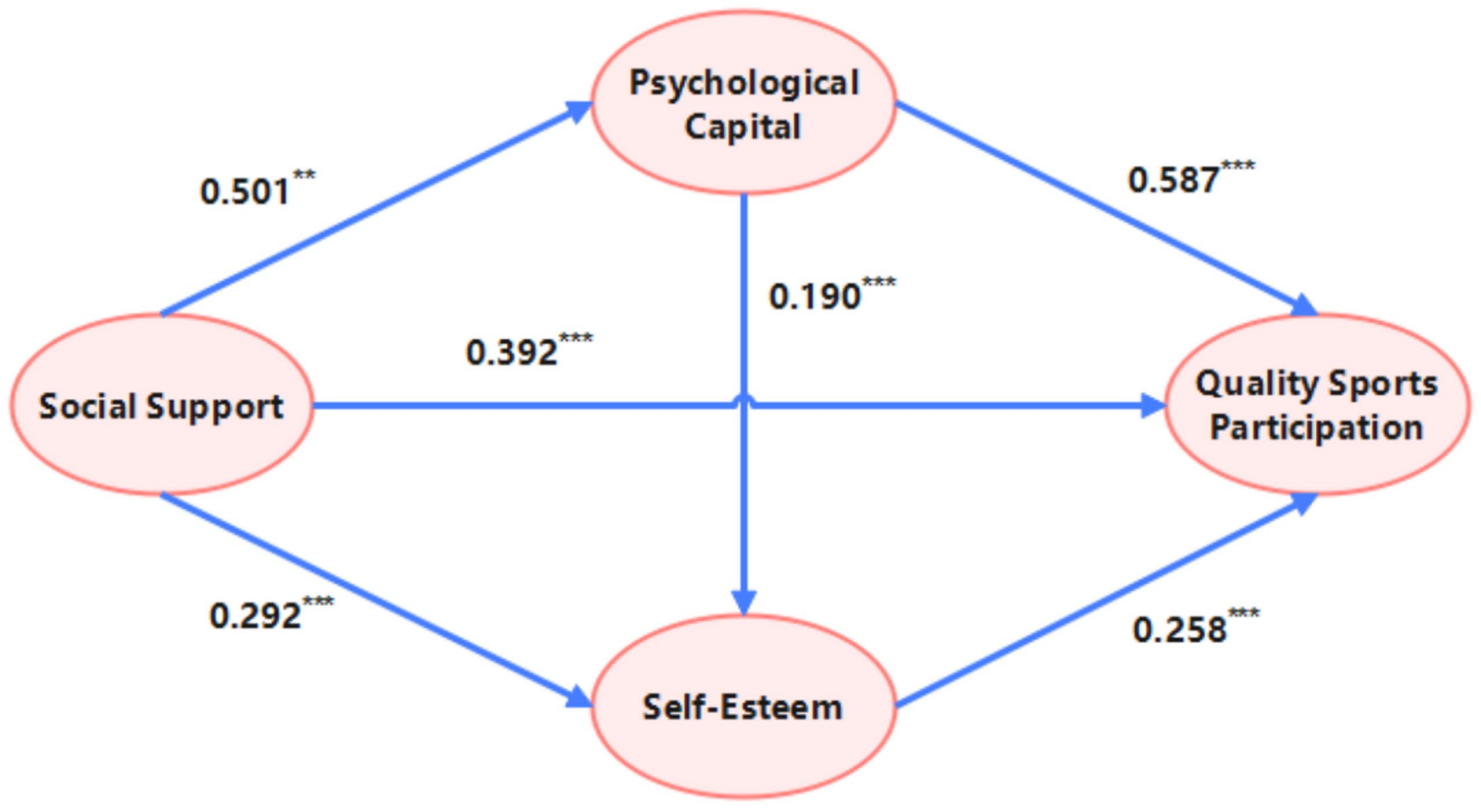
Chain mediation model of social support and quality of sports participation.

Decomposition of the total indirect effect into specific paths showed: Path 1 (SS → PC → QSP): The indirect effect was 0.294 (SE = 0.042), significant (95% CI [0.217, 0.384]), accounting for 37.41% of the total effect. This was the strongest mediating path; Path 2 (SS → SE → QSP): The indirect effect was 0.075 (SE = 0.027), significant (95% CI [0.026, 0.133]), accounting for 9.54% of the total effect; Path 3 (SS → PC → SE → QSP): The chain mediating effect was 0.025 (SE = 0.010), significant (95% CI [0.008, 0.048]), accounting for 3.18% of the total effect (Figure 2).

## 5 Discussion

This study, through theoretical construction and empirical validation, reveals the impact of SS on the QSP among individuals with disabilities and its underlying mechanism. The chain mediation pathway involving PC and SE provides a crucial explanatory framework for understanding how external environmental support is internalized into sustained intrinsic motivation for sports participation. This finding shifts the research perspective from merely focusing on the provision of SS to the psychological processing and transformation mechanisms individuals with disabilities employ regarding external resources, thereby deepening the theoretical implications of the “Environment-Individual-Behavior” interaction model within the context of sports participation for this specific population.

The facilitative role of SS in the sports participation of individuals with disabilities must be interpreted against the backdrop of the unique challenges they face. Compared to the general population, individuals with disabilities often contend with multiple constraints, including functional limitations, environmental barriers, and potential social stigmatization. In this context, the value of SS extends beyond providing instrumental assistance; it crucially conveys signals of social acceptance and affirmation of capability (Zhang, 2025). This social validation functions by reshaping the individual’s psychological resource system.

PC, as the core component of the mediation mechanism, demonstrates its importance by constituting the “psychological toolkit” for individuals with disabilities to confront athletic challenges. When individuals perceive support from family, peers, or the community, this positive external input is first transformed into enhanced self-efficacy, resilience, optimism, and hope regarding their coping abilities (Deng and Wang, 2024). These positive psychological traits enable individuals to proactively translate SS into concrete behavioral strategies.

SE’s position within the chain highlights the value of sports participation in reconstructing the self-concept of individuals with disabilities. While SS can directly offer affirmation of worth, its deeper impact on SE relies more heavily on the “proof of capability” accumulated through PC. Skill advancement, overcoming difficulties, or contributions made within a team during sports activities provide tangible achievements that form the empirical basis for enhanced SE (Moon and Kim, 2021). PC acts as an “achievement catalyst” in this process: high self-efficacy prompts individuals to set appropriately challenging goals, resilience ensures persistence through setbacks, optimism maintains positive affect, and hope provides pathway planning toward goal attainment. Each successful sports experience reinforces the “I can do it” cognition, gradually dismantling negative self-schemas formed by disability or social prejudice, and ultimately internalizing a stable sense of self-worth. This enhancement of SE, rooted in demonstrated competence, possesses greater resilience and durability compared to mere external affirmation, and is more potent in stimulating intrinsic motivation for deep engagement. Therefore, the transformation from PC to SE essentially completes the psychological leap from “I can participate” to “My participation has value.”

The significant chain pathway “SS → PC → SE → QSP” reveals the hierarchical progression and synergistic gain effect of positive psychological resources. This chain indicates that the influence of external support on behavior necessitates a complete psychological processing sequence: “resource internalization → capability construction → value confirmation.” PC constitutes the foundational layer of psychological resources, providing direct impetus for individuals to tackle the practical challenges of sports participation. SE, in contrast, represents a deeper motivational and value system, imbuing participation with a sense of meaning and persistence. These two are not simply parallel; they exhibit sequential dependence. Stable SE requires a foundation of actual competence experiences, and PC serves as the psychological engine ensuring the continuous generation of such experiences. This hierarchical nature explains why, although the chain pathway’s effect size is relatively small, it holds critical theoretical significance. It delineates the complete psychological process necessary for SS to ultimately translate into high-quality, sustainable participation behavior. For example, an amputee runner receiving support from a professional coach (SS) first enhances their training capabilities and competition confidence (PC). Completing races then fosters a sense of achievement and strengthens their “runner” identity (SE), ultimately leading to a stable behavioral pattern of persevering through difficulties and proactively improving training quality (participation quality). A break in any link of this chain can lead to diminished effectiveness of the support.

In conclusion, by uncovering the chain mediating roles of PC and SE, this study elucidates the deep-seated psychological mechanisms through which SS enhances the QSP among individuals with disabilities. It emphasizes that high-quality sports participation is not merely about improved physical conditions or increased behavioral frequency. Rather, it is fundamentally a psychological reconstruction process whereby external support, through the stepwise transformation via individual positive psychological resources, is ultimately internalized into stable value identification and behavioral patterns. This theoretical advancement lays a solid foundation for constructing a paradigm for promoting sports participation among individuals with disabilities that balances environmental adaptability and psychological agency.

## 6 Conclusions and prospects

### 6.1 Research conclusions

Based on a stratified random sampling survey of 632 individuals with disabilities across 12 cities in 6 provinces spanning Eastern, Central, and Western China, this study thoroughly investigated the mechanism by which SS influences the QSP among individuals with disabilities, with a specific focus on testing the chain mediating roles of PC and SE. The results confirm that SS not only exerts a significant and direct positive effect on the QSP but also indirectly enhances it through two crucial psychological pathways involving PC and SE. Specifically, SS effectively boosts the PC level of individuals with disabilities. This enhanced PC not only directly predicts higher levels of sports participation behavior and quality but also further cultivates individual SE. SE itself also exerts an independent positive influence on the QSP.

### 6.2 Research limitations

While this study has made progress in exploring the impact of SS on the QSP among individuals with disabilities and its underlying psychological mechanisms, several noteworthy limitations exist and should be addressed in future research.

Sample Representativeness: Although 632 valid questionnaires were obtained from 12 cities and the sample size met statistical power requirements, participants were primarily aged 20 to 60 years. The sports participation experiences of adolescents and elderly individuals with disabilities were insufficiently represented. Furthermore, while urban–rural distribution was considered, more detailed stratification or control for key demographic variables such as type of disability, severity of disability, and time since disability onset was lacking. This may limit the generalizability of the findings to different subgroups within the disability population.

Cross-Sectional Design: The use of a cross-sectional survey design makes it difficult to establish definitive causal relationships between variables. Although the theoretical model and statistical analyses support the directionality of the “SS → PC/SE → QSP” pathway, cross-sectional data cannot fully rule out the possibility of reverse causality or the influence of third variables. Future research should employ longitudinal tracking or experimental designs to further validate the temporal sequence and causal chains among variables.

Self-Report Measures and Potential Biases: The measurement of core variables relied primarily on self-reported scales, which may be susceptible to common method bias and social desirability effects. Although Harman’s single-factor test and questionnaire design measures indicated that common method bias was not severe, the subjective nature of self-reported data for all variables may have interfered with the estimated strength of the relationships between variables.

Measurement of QSP: The dimensionality of measuring QSP warrants expansion. The Physical Activity Rating Scale (PARS-3) used in this study primarily assessed exercise intensity, frequency, and duration. While these are important indicators of participation volume, the connotation of “participation quality” may be richer. The current measurement may not have comprehensively captured its multidimensional attributes (e.g., subjective experiences like enjoyment, autonomy, social connection, sense of competence).

Potential Omitted Variables and Model Scope: The model construction potentially suffers from omitted variable bias. The study focused on the chain mediating roles of PC and SE, confirming their importance. However, sports participation behavior among individuals with disabilities is inevitably influenced by a wider array of internal and external factors. Variables not included in the model (e.g., physical accessibility, perceived discrimination, access to rehabilitation services, specific motivations, economic constraints, sport type characteristics) may be significant covariates or moderators affecting the outcomes. Finally, while the theoretical model verified the chain mediation effect, the dynamic relationship between PC and SE, as well as the differential pathways of specific dimensions of SS (e.g., emotional vs. instrumental), were not explored in depth, limiting the nuanced depiction of the theoretical mechanism.

### 6.3 Future prospects

#### 6.3.1 Theoretical level

Exploring Complex Dynamics: Future research should delve deeper into the more complex dynamic relationships between PC and SE, and their boundary conditions. While this study validated the “SS → PC → SE” chain, potential bidirectional influences, the specific mechanisms linking different dimensions of PC to SE, and the moderating effects of individual characteristics or environmental factors on these pathways warrant further investigation to build a more refined theoretical model. In addition, it is suggested that subsequent studies can introduce the type and degree of disability as moderating variables based on this model to deepen the understanding of differences within the group.

Integrating Diverse Factors: It is necessary to expand the model to incorporate a more diverse range of influencing factors, constructing a more comprehensive explanatory framework. Future theoretical models should consider integrating other potential key variables, such as accessible physical environments, perceived social discrimination, accessibility of rehabilitation services, specific motivation types, economic resource constraints, and characteristics of different sports types. Methods like structural equation modeling or multilevel analysis could be used to clarify how these factors interact with SS, PC, and SE to collectively shape the QSP for individuals with disabilities, thereby enhancing the ecological validity and explanatory power of the theory.

Developing Multidimensional Assessment Tools: Efforts should be directed toward developing and refining multidimensional assessment tools specifically suitable for evaluating QSP among individuals with disabilities. Given the current measurement’s focus on exercise volume, future research needs to develop or validate comprehensive evaluation systems that include dimensions of subjective experience (e.g., enjoyment, satisfaction, autonomy, relatedness, perceived competence) and objective functional indicators, to capture the rich connotation of “participation quality” more comprehensively and accurately, providing a more solid measurement foundation for theoretical construction.

#### 6.3.2 Practical level

Developing Structured Psychological Interventions: Based on the core mediating mechanisms, future practice should design and validate structured psychological promotion programs for individuals with disabilities. This includes developing PC enhancement courses and SE activation interventions, exploring their feasibility and effectiveness when integrated into existing sports services for individuals with disabilities. The aim is to amplify the positive effect of SS on sports participation by strengthening internal psychological resources.

Building Multilevel, Targeted Support Networks: Practical interventions should strive to build multi-level (micro, meso, macro) and precisely targeted SS networks to empower sports participation among individuals with disabilities. Efforts need to move beyond general advocacy for support and instead provide differentiated support content tailored to the specific needs of various disability types, age groups, and urban/rural backgrounds across these levels, ensuring the effective delivery and perception of support resources.

Implementing and Evaluating Integrated Interventions: It is recommended to promote the establishment of integrated intervention models combining “SS – Psychological Resources – Behavioral Participation” and conduct long-term effectiveness evaluations. Future practical exploration should design comprehensive projects that combine the creation of supportive external environments with the cultivation of internal PC and SE, informed by the chain mechanism revealed in this study. Through rigorous randomized controlled trials or quasi-experimental designs, the short-term effects and long-term benefits of such interventions should be tracked. This will provide evidence-based guidance for policy formulation and service optimization, ultimately fostering a virtuous cycle where theory guides practice and practice informs theory.

## Data Availability

The original contributions presented in the study are included in the article/Supplementary material, further inquiries can be directed to the corresponding author.
